# Level-Set Segmentation-Based Respiratory Volume Estimation Using a Depth Camera

**DOI:** 10.1109/JBHI.2018.2870859

**Published:** 2018-09-28

**Authors:** KyeongTaek Oh, Cheung Soo Shin, Jeongmin Kim, Sun K. Yoo

**Affiliations:** 1 Department of Medical EngineeringYonsei University College of Medicine 200355 Seoul03722South Korea; 2 Department of Anesthesiology and Pain Medicine, Gangnam Severance HospitalYonsei University College of Medicine 200355 Seoul03722South Korea; 3 Department of Anesthesiology and Pain Medicine, Severance HospitalYonsei University College of Medicine 200355 Seoul03722South Korea

**Keywords:** Respiratory volume, level-set method, depth camera, non-contact, ventilator

## Abstract

In this paper, a method is proposed to measure human respiratory volume using a depth camera. The level-set segmentation method, combined with spatial and temporal information, was used to measure respiratory volume accurately. The shape of the human chest wall was used as spatial information. As temporal information, the segmentation result from the previous frame in the time-aligned depth image was used. The results of the proposed method were verified using a ventilator. The proposed method was also compared with other level-set methods. The result showed that the mean tidal volume error of the proposed method was 8.41% compared to the actual tidal volume. This was calculated to have less error than with two other methods: the level-set method with spatial information (14.34%) and the level-set method with temporal information (10.93%). The difference between these methods of tidal volume error was statistically significant ${\text{(p}} < {\text{0.0001}})$. The intra-class correlation coefficient (ICC) of the respiratory volume waveform measured by a ventilator and by the proposed method was 0.893 on an average, while the ICC between the ventilator and the other methods were 0.837 and 0.879 on an average.

## I. Introduction

Breathing is a critically important human physiological activity. Respiration volume has been used as an important indication in the diagnosis and treatment of pulmonary diseases that require rapid medical diagnosis and attentive care. Therefore, it is important to measure respiratory volume and evaluate pulmonary functions effectively. There have been numerous studies on methods for measuring respiratory volume using, for example, electrical impedance tomography [1], body plethysmography [2], and strain gauges [3]. However, these methods require contact with the human body and may interfere with respiration by causing a sense of physical limitation or a feeling of irritation. To overcome these inconveniences, several methods have been studied by which to measure the respiratory volume without the need for contact [4]–[6]. Among the non-contact methods, the use of depth cameras has several advantages. This method is capable of assessing regional pulmonary function from asymmetrical movements of the chest wall. Convenient installation allows measurement in a variety of environments, and the cost of this method is low.

When calculating the respiratory volume using a depth camera, it is important to distinguish the respiration-related region in the depth image. In previous studies using depth cameras, predefined regions [7] or joint points [8] were applied to distinguish the respiration-related region. The respiratory volume was estimated by multiplying the difference of the depth value by the actual size of the distinguished respiration-related region.

In this paper, a method is proposed by which to measure the human respiratory volume from morphological changes of the chest wall by distinguishing only the respiration-related region using the level-set method. The result from estimation of respiratory volume using this method were verified by comparing them with the respiratory volume measured with a ventilator. To show that the proposed method outperforms the two other non-contact methods presented in this paper, we compared the accuracy of the respiratory volumes calculated by the various methods.

## II. Methods

### A. Experimental Setup

#### 1) Experimental Settings

**Fig. 1.**
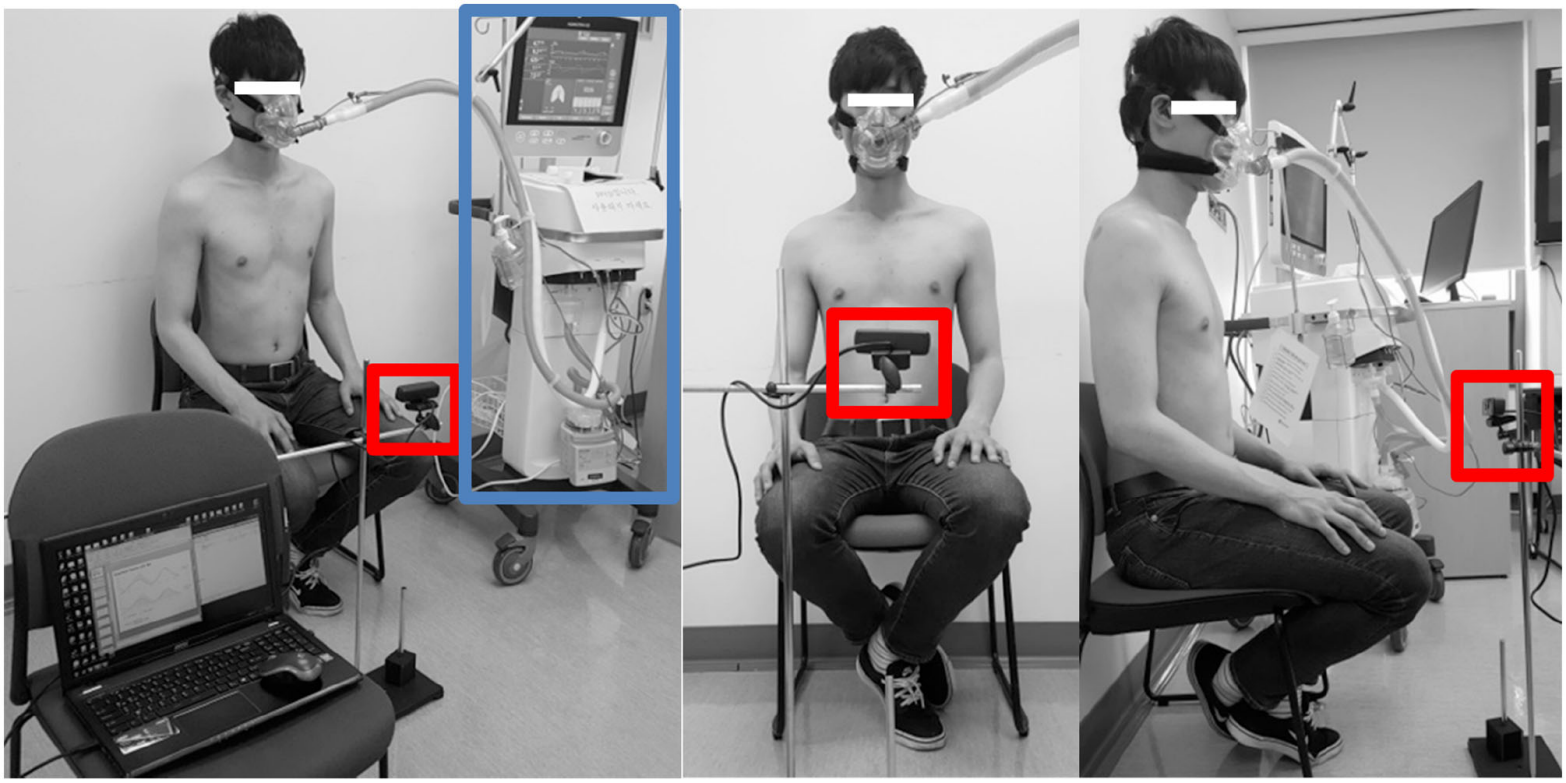
Experimental environment (red box: depth camera, blue box: ventilator).

Respiratory activity was recorded independently by a depth camera (Creative Sez3D) [9] and by a ventilator. A sequence of separate depth images was recorded with associated time stamps at 16 frames per second (fps). The depth image resolution was ${\text{320}}\times {\text{240 pixels}}$. Fig. 1 shows the experimental environment. In Fig. 1, the red box shows the depth camera and blue box shows the ventilator. In experiments, subjects were instructed to breathe as much air as was coming from a ventilator for two minutes and to look at the front of the camera. As the amount of air pumped from the ventilator changed, the number of breath in 2 minutes varied from 25 to 35. The experiments were repeated three times while changing the input volume from the ventilator per subject. The distance between the camera and the subject ranged from 40 to 50 cm. The output of the ventilator was used as a standard to evaluate the respiratory volume. The ventilator used in the experiment was a Hamilton G5 (Hamilton Medical AG) [10].

#### 2) Clinical Settings

For the experiment, 10 male subjects $({\rm{age }} = 25.1 \pm 1.3{\ \rm{y}},{\rm{ BMI }} = {\text{22.54}} \pm {\text{2.02}}{\ \text{kg}}/{{\text{m}}^2},$ chest circumference $= {\text{90.4}} \pm \text{4.9}\, \text{cm},$ abdominal circumference $= {\text{77.9}} \pm {\text{5.8}}{\ \text{cm}})$ without lung disease were recruited. Table I shows the information about the subjects. The experiment in this study was performed at Severance Hospital and was approved by the Institutional Research Board of Severance Hospital (reference number 1-2015-0054). The subjects voluntarily agreed prior to the experiment to participate in this clinical study and written informed consent was obtained from each participant.

**TABLE I:** Overview of Information About Participants in this Study

Subjects	Gender	Age[years]	${\rm{BMI[kg/}}{{\rm{m}}^{2}}{{]}}$	CC[cm]	AC[cm]
1	M	25	24.1	96	86
2	M	26	22.4	93	76
3	M	24	18.94	89	68
4	M	23	23.46	95	82
5	M	27	20.38	88	75
6	M	27	22.39	86	80
7	M	25	21.36	80	73
8	M	24	24.58	95	85
9	M	25	22.09	89	73
10	M	25	25.73	93	81
Mean		25.1	22.54	90.4	77.9
STD		1.3	2.02	4.9	5.8

^*^CC: Chest circumference, AC: Abdominal circumference, STD: Standard deviation

### B. Respiration-Related Region

The main body parts involved in respiration include the airways, the lungs, the blood vessels connected to the lungs, and the muscles involved in respiration [11]. Among these body parts, respiratory muscles cause changes that can be observed with a camera. The respiratory muscles consist of the diaphragm, the intercostal muscles, and the muscles that support breathing [12]. During inspiration, the chest wall and cavity expand due to contraction of the intercostal muscles and the diaphragm. When a person inhales air, intercostal muscles between the ribs enlarge the chest cavity. The dome-shaped diaphragm contracts downward and becomes flat. The flattened diaphragm pushes the viscera and produces a visible outward movement of the abdominal wall. During expiration, the intercostal muscles between the ribs relax, which causes the chest cavity to compress. The diaphragm also relaxes and moves upward into the chest cavity. This returns the viscera to their original position and retracts the abdominal wall. For this paper, the respiration-related region was defined as the region of the chest wall that shows an outward bulge due to the action of the intercostal muscles and diaphragm.

### C. Level-Set Method

The level-set method was used to distinguish only the respiration-related region. In the sequence of depth images, spatial and temporal information was combined with the level-set method. The shape of the human chest wall was used as spatial information. As temporal information, the respiration-related region segmented from the previous frame in a time-aligned depth image was used.

#### 1) Spatial Information

While breathing, the chest wall moves in a pattern related to respiration by the muscles associated with breathing. In contrast, body parts other than the chest wall, such as arms, have motions unrelated to respiration. Therefore, regions showing non-respiratory movement need to be excluded. Traditional level-set methods such as the Chan-Vese method [13] are often used in image segmentation methods. Nevertheless, traditional segmentations tend to fail when images are corrupted or when object boundaries are ambiguous. In the depth image, there is a limitation in distinguishing the chest wall region because the depth values of the chest wall and the arm regions are similar. To solve this problem, level-set segmentation was used along with the shape-prior knowledge method [14] proposed by C. Arrieta. This method adds an energy term for shape-prior knowledge to the Chan-Vese method. This energy term forces the curve to separate properly, these ambiguous boundaries by measuring dissimilarity in the shape from prior knowledge and the evolving curve. The dissimilarity of the shape can be calculated using (1).

\begin{align*}
{E_{shape}} = {d^2}(\phi,{\phi _0}) =& \int\nolimits_{\Omega }({H}\left(\phi \left({Q^T}\Lambda \vec{x} + M\right)\right)\nonumber\\
& - H({\phi _0}(\vec{x})))^2 d\vec{x}\tag{1}
\end{align*}

where $\vec{x}$ is a location vector, and $\phi $ and ${\phi _0}$ are signed distance functions of the evolving curve and a shape prior, respectively. $H({\phi ({\vec{x}})})$ is the Heaviside function of $\phi ({\vec{x}})$, $\boldsymbol{\Omega} $ is the image domain, and $Q,\lambda $, and $M$ are the eigenvectors, eigenvalues, and center of gravity of $\phi $. In (1), the dissimilarity is measured in the normalized coordinate system by rotating, translating, and scaling. To obtain the evolution equations, we need to calculate the derivative of (1) with respect to $\phi $, which can be expressed as (2).

\begin{align*}
&{\left. {\frac{{\partial {E_{shape}}(\phi)}}{{\partial \phi }}} \right|_{\phi (\vec{x})}} \nonumber\\
& \quad = \int\nolimits_{\Omega }{{2\delta (\phi)D(\phi,{\phi _0})\left| {\det ({\Lambda ^{ - 1/2}})} \right|\tilde{\phi }(\vec{x})d\vec{x}}} \nonumber\\
& \quad \quad+\int\nolimits_{\Omega }{{2D(\phi,{\phi _0})\delta (\phi)\nabla \varphi \vec{x}}}\nonumber\\
& \quad \quad\times\int\nolimits_{\Omega }{{\frac{{\partial Q}}{{\partial \phi }}\tilde{\phi }(\vec{x})d\vec{x}{Q^T}(\vec{x} - M)\left| {\det ({\Lambda ^{ - 1/2}})} \right|d\vec{x}}} \nonumber\\
& \quad \quad+ \int\nolimits_{\Omega }{{D(\phi,{\phi _0})\delta (\phi)\nabla \phi \vec{x}Q{\Lambda ^{ - 1/2}}}}\nonumber\\
& \quad \quad\times\int\nolimits_{\Omega }{{\frac{{\partial \Lambda }}{{\partial \phi }}\tilde{\phi }(\vec{x})d\vec{x}{\Lambda ^{ - 1/2}}{Q^T}(\vec{x} - M)\left| {\det ({\Lambda ^{ - 1/2}})} \right|d\vec{x}}} \nonumber\\
& \quad \quad+ \int\nolimits_{\Omega }{{2D(\phi,{\phi _0})\delta (\phi)\nabla \phi \vec{x}}}\nonumber\\
& \quad \quad\times\int\nolimits_{\Omega }{{\frac{{\partial M}}{{\partial \phi }}\tilde{\phi }(\vec{x})d\vec{x}\left| {\det ({\Lambda ^{ - 1/2}})} \right|d\vec{x}}} \tag{2}
\end{align*}

where δ is the Dirac delta function and $D({\phi,{\phi _0}})$ is defined in (3).

\begin{equation*}
D(\phi,{\phi _0}) = H(\phi (\vec{x})) - H({\phi _0}({Q^T}\Lambda \vec{x} + M)))\tag{3}
\end{equation*}

This method was used to distinguish the chest wall region in the depth image. As shape-prior knowledge, a manually separated chest-wall shape was used and described as spatial information.

#### 2) Temporal Information

Movement of the chest wall while breathing causes changes observable with a depth camera. These are indicated as changes in the depth value over time. However, changes in depth value that are unrelated to respiration also appear. The location where the depth change occurs gives information to distinguish the respiration-related region. It is suitable for use in respiration probability estimation. For instance, if the location of the depth change is in the chest wall region, it is likely that the change is caused by respiration activity. Therefore, we define the respiration-region information function with (4).

\begin{equation*}
p(\vec{x}) = \left\{ {\begin{array}{cc}
{1,} & {\vec{x} \in W}\\
{H(\phi (\vec{x})),}&{\text{otherwise}} \end{array}} \right.\tag{4}
\end{equation*}

where $W$ is the chest wall region that is the result of the shape prior level-set method. When a depth change occurs outside the chest wall region, $p({\vec{x}})$ is decreased by the Heaviside function. This means that if depth change occurs far away from the chest wall region, the probability that this change is caused by respiratory activity gets low.

Using the level-set method with the region information was proposed by Gang [15]. Like the shape prior level-set, this method adds an energy term to the region information function, which is defined in (5).

\begin{equation*}
{E_{region}} = - \iint\nolimits_{IF} {p(\vec{x})d\vec{x}}\tag{5}
\end{equation*}

where $IF$ is the interior field of the curve. The integral in (5) is used to find the boundary of the curve where ${E_{region}}$ is minimized. To obtain the evolution equations, we need to calculate the derivative of (5). The derivation result is expressed in (6).

\begin{equation*}
\frac{{\partial {E_{region}}}}{{\partial t}} = p(\vec{x}) \cdot \vec{N},\vec{N} = \left(\frac{{dy}}{{ds}}, - \frac{{dx}}{{ds}}\right)\tag{6}
\end{equation*}

where $\vec{N}$ is the outer vector that represents the evolution direction of the curve, and $s$ is the arc length parameter. This method attempts to distinguish depth changes only in the respiration-related region. Therefore, the region based level-set method is performed on the difference depth image. This image can be obtained by calculating the difference between the depth image of the current frame and the previous frame.

#### 3) Adaptive Function Modeling

The respiration-related region continuously grows larger and smaller in response to the respiration. If camera noise or movement other than respiration is present, there will be a change for a short period and this change will appear in a discontinuous location in the depth difference image. Based on these characteristics, an adaptive region information function is proposed to distinguish the respiration-related region in the current frame by weighting the region that was segmented as the respiration-related region in the previous frame. This function is defined in (7).

\begin{align*}
{p_a}(\vec{x}) &= \left\{ {\begin{array}{cc}
{1 + f(\vec{x}),} & {\vec{x} \in {W_t}}\\
{H(\phi (\vec{x})),}&{\text{otherwise}} \end{array}} \right.\nonumber\\
f(\vec{x})& = \left\{ {\begin{array}{cc}
{TH,}&{\vec{x} \in {R_{t - 1}},{\phi _{t - 1}}(\vec{x}) \geq TH}\\
{{\phi _{t - 1}}(\vec{x}),}&{\vec{x} \in {R_{t - 1}},0 \leq {\phi _{t - 1}}(\vec{x}) \leq TH}\\
{0,}&{{\phi _{t - 1}}(\vec{x}) \leq 0} \end{array}} \right. \tag{7}
\end{align*}

where ${R_{t - 1}}$ is the segmented respiration-related region of the previous frame and ${\phi _{t - 1}}$ is the signed distance function of ${R_{t - 1}}$. Here, $f({\vec{x}})$ is a function that transfers the weight of the result of the previous frame. In $f({\vec{x}})$, a threshold was applied to avoid repeatedly adding large weights to a specific region. In this paper, the threshold was set to ‘1’ for all subjects. This adaptive region information function was applied from the second iteration in the region-based level-set method.

### D. Respiratory Volume Estimation

After distinguishing the respiration-related region, the respiratory volume can be calculated based upon the respiration-related region. In the sequence of depth images, the location of the pixels, $\vec{r},$ within the respiration-related region is determined in each depth image. The volume change can be obtained by calculating the difference of the current depth image and the previous depth image. The difference volume ${\rm{V}}$ at a specific point in time can be obtained using (8).

\begin{equation*}
V = \sum\limits_{\vec{d} \in R} {(U(\vec{r}) - L(\vec{r})) \times C{{(\vec{r})}^2}}\tag{8}
\end{equation*}

where U and L is the current and previous frame of the depth image, respectively. C is the coefficient that translates the measurement unit from pixel number to actual length (mm). This coefficient is represented by the following linear function.

**Fig. 2.**
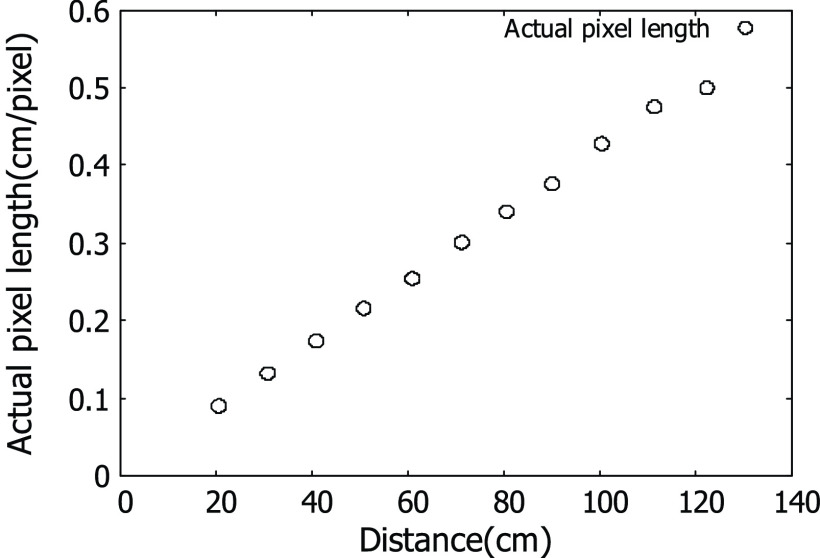
Relationship between distance and actual pixel length.

\begin{equation*}
{\rm{C}\ }\left({\mathop{x}\limits^{\rightharpoonup}} \right) = 0.0041 \times {\rm{d}}\left({\mathop{x}\limits^{\rightharpoonup}} \right) + 0.0046\tag{9}
\end{equation*}

where d is the depth value for the pixel location $\vec{x}$. As shown in equation (9), the coefficient C is selected according to the depth value for each pixel in the separated respiration-related region. Equation (9) represents the relationship between the sensing distance and the actual pixel length. The relationship between these two variables can be obtained by dividing the number of pixels corresponding to the region of the fixed size object in the depth image while changing the sensing distance. The experimental results for the relationship between these two variables are shown in Fig. 2. In Fig. 2, it can be seen that the actual pixel size increases with distance.

If the length of the depth frame acquired in the volume measurement is ${\rm{k}}$, the volume difference for each depth image forms a separate time series $\{ {{\rm{V}}({\rm{t}})} \}_{t=1}^k$ of length ${\rm{k}}$. In this paper, the series of difference volumes $\{ {{\rm{V}}({\rm{t}})}_{t= 1}^k$ was defined as the respiratory volume waveform. The respiratory volume in each breath is estimated by detecting the peak and valley points of the respiratory volume waveform.

### E. Evaluation Parameters

#### 1) Tidal Volume Error

Tidal volume error can be calculated using equation (10). In equation (10), $VD$ and ${\rm{VV}}$ are the respiratory volume of each breath corresponding to the respiratory volume waveform calculated from the method presented in this paper and the respiratory volume waveform obtained from a ventilator. Index ${\rm{i}}$ represents the index corresponding to each breath in the respiratory volume waveform. As shown in equation (10), the tidal volume error can be obtained by calculating the difference in respiratory volume for each breath and then calculating the average of the differences.

\begin{equation*}
{\text{Tidal}}\; {\text{Volume}} \;{\text{Error}} = \frac{{\mathop \sum \nolimits_{i\ = \ 1}^{number\ of\ breaths} \frac{{\left| {V{D_i} - V{V_i}} \right|}}{{V{V_i}}} \times 100}}{{\text{number}}\ {\text{of}}\ {\text{breaths}}}\tag{10}
\end{equation*}

#### 2) Volume Waveform Error

Volume waveform error can be calculated using equation (11). In equation (11), ${\rm{WD}}$ and ${\rm{WV}}$ are the respiratory volume waveform calculated from the method presented in this paper and that from the ventilator. ${\rm{T}}$ and ${\rm{t}}$ denote the total time length of the respiratory volume waveform and the index of the specific time point, respectively. As shown in equation (11), the volume waveform error is obtained by subtracting the difference in the waveform at each time point and calculating their summation. In this paper, 2-minute length respiratory volume waveforms obtained from the experiment were used to calculate the respiratory volume waveform error.

\begin{equation*}
{\text{Volume}}\ {\text{Waveform}}\ {\text{Error}} = \frac{{\mathop \sum \nolimits_{t\ = \ 0}^T \left| {W{D_t} - W{V_t}} \right|}}{{\mathop \sum \nolimits_{t\ = \ 0}^T \left| {W{V_t}} \right|}} \times 100\tag{11}
\end{equation*}

#### 3) Intra-Class Correlation

The model used to calculate the ICC was the 2-way fixed effects model (among the 10 ICC models defined by McGraw and Wong [16]). This model was chosen because all of the experimental data was acquired using the same equipments and using specific equipment (the ventilator and depth camera). Moreover, the ICC definition was selected as absolute agreement because ICC is calculated to see how closely the volume waveform acquired from a ventilator matches the volume waveform of the method proposed in this paper. The ICC can be calculated using equation (12). In equation (12), $M{S_R}$ and $M{S_c}$ represent the mean square between two volume waveforms to be compared for each time point and mean square within each waveform. $M{S_E}$ indicates the mean square for the error between the two volume waveforms. In this case, ${\rm{k}}$ and ${\rm{n}}$ represent the number of volume waveforms to be compared and the number of samples of the waveform, respectively.

\begin{equation*}
{\rm{ICC}\ } = \frac{{M{S_R} - M{S_E}}}{{M{S_R} + \left({k - 1} \right) \times M{S_E} + \frac{k}{n} \times \left({M{S_C} - M{S_E}} \right)}}\tag{12}
\end{equation*}

## III. Results

We measured the respiratory volumes of 10 subjects with a ventilator and a depth camera. To evaluate the respiratory volume measurement system, the segmentation result using spatial information and time information was compared with the traditional method in the spatial domain. In addition, the respiratory volume waveforms obtained by the proposed method and by the other methods presented in this paper were compared to the waveform of the ventilator. A validation test was performed to verify the accuracy of the respiratory volume.

### A. Level-Set Method With Spatial Information

**Fig. 3.**
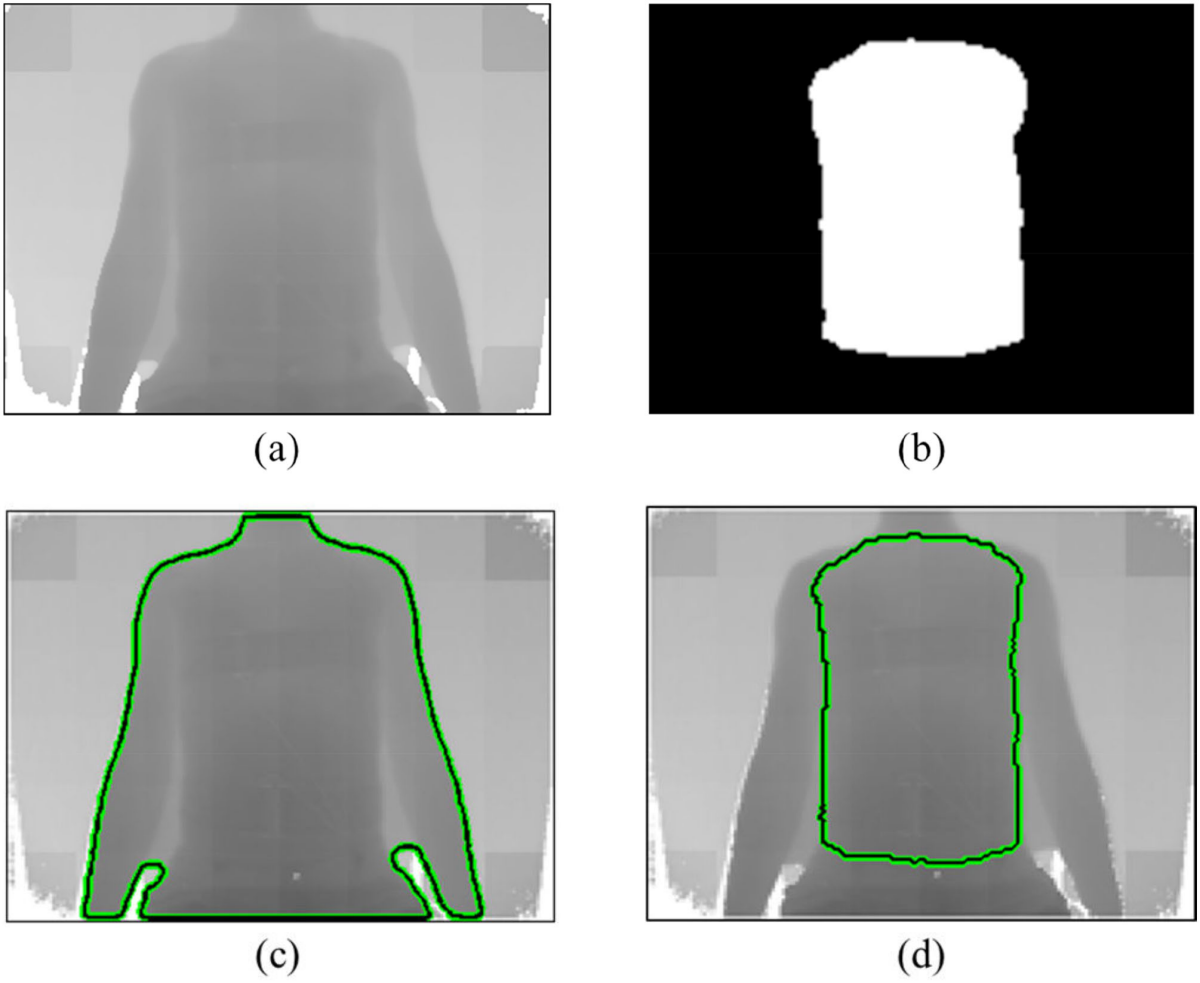
Segmentation result for the chest wall region in a depth image. (a) Depth image of human body. (b) Shape prior. (c) Chan-Vese level-set segmentation result. (d) Shape prior level-set segmentation result.

Fig. 3 shows the segmentation result of the chest wall region in a depth image. On the upper body of a human, it can be seen that the boundary between regions is ambiguous because the depth values of the chest wall region and the arm region are similar, as can be seen in Fig. 3(a). Due to the ambiguous boundaries, the conventional Chan-Vese level-set method separates the entire upper body including the region shown in Fig. 3(c). On the other hand, the level-set method using shape information can separate the chest wall region despite the ambiguous boundaries, as shown in Fig. 3(d).

### B. Level-Set Method With Temporal Information

**Fig. 4.**
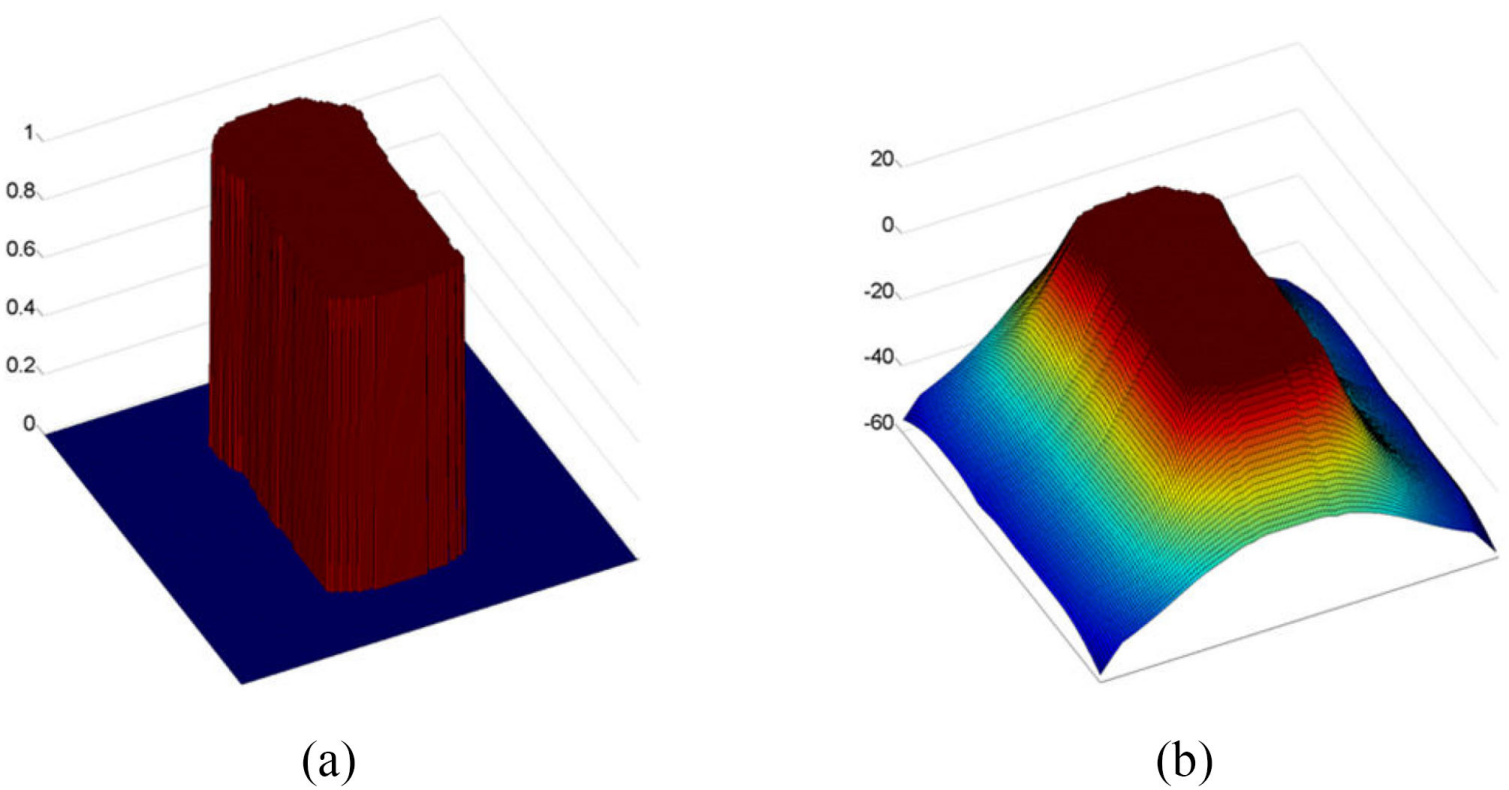
Visualization of the region information function. (a) Segmented chest wall region. (b) Region information function ${{\bf p}}({{\boldsymbol{\vec{x}}}})$.

The level-set method with temporal information uses the region information function. This function uses the segmented chest wall region shown in Fig. 4(a). In Fig. 4(b), this function generates a force that causes the depth change to be regarded as a respiration-related region if the changing point is within the chest wall region. On the other hand, if the changing point is far away from the boundary of the chest wall region, the opposing force is strengthened.

**Fig. 5.**
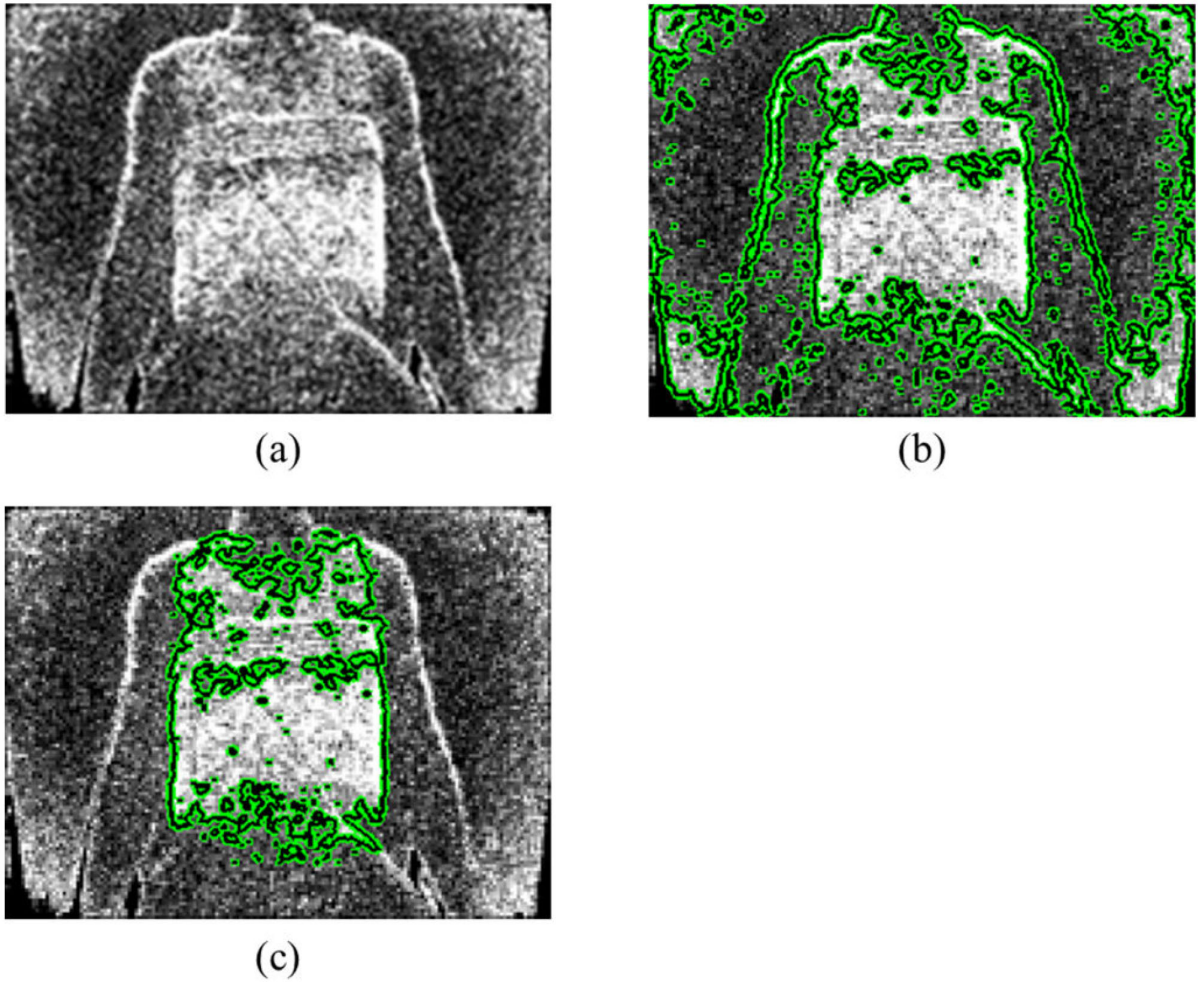
Segmentation result of respiration related-region. (a) Difference depth image. (b) Chan-Vese level-set segmentation result. (c) Region-based level-set segmentation result.

To distinguish the respiration-related region where the depth value actually changes, the respiration-related region was separated in the difference depth image. The difference depth image is shown in Fig. 5(a). The brighter pixel intensity means that there was strong motion in the difference depth image. In Fig. 5(a), it can be seen that strong motion occurred in the chest wall region, the arm boundary, and at the corners of the difference depth image. The conventional Chan-Vese level-set method separates all the depth changes in the difference image as shown in Fig. 4(b). On the other hand, the region-based level-set separates only the depth change in the chest wall region, as shown in Fig 4(c).

### C. Level-Set Method With Adaptive Function

**Fig. 6.**
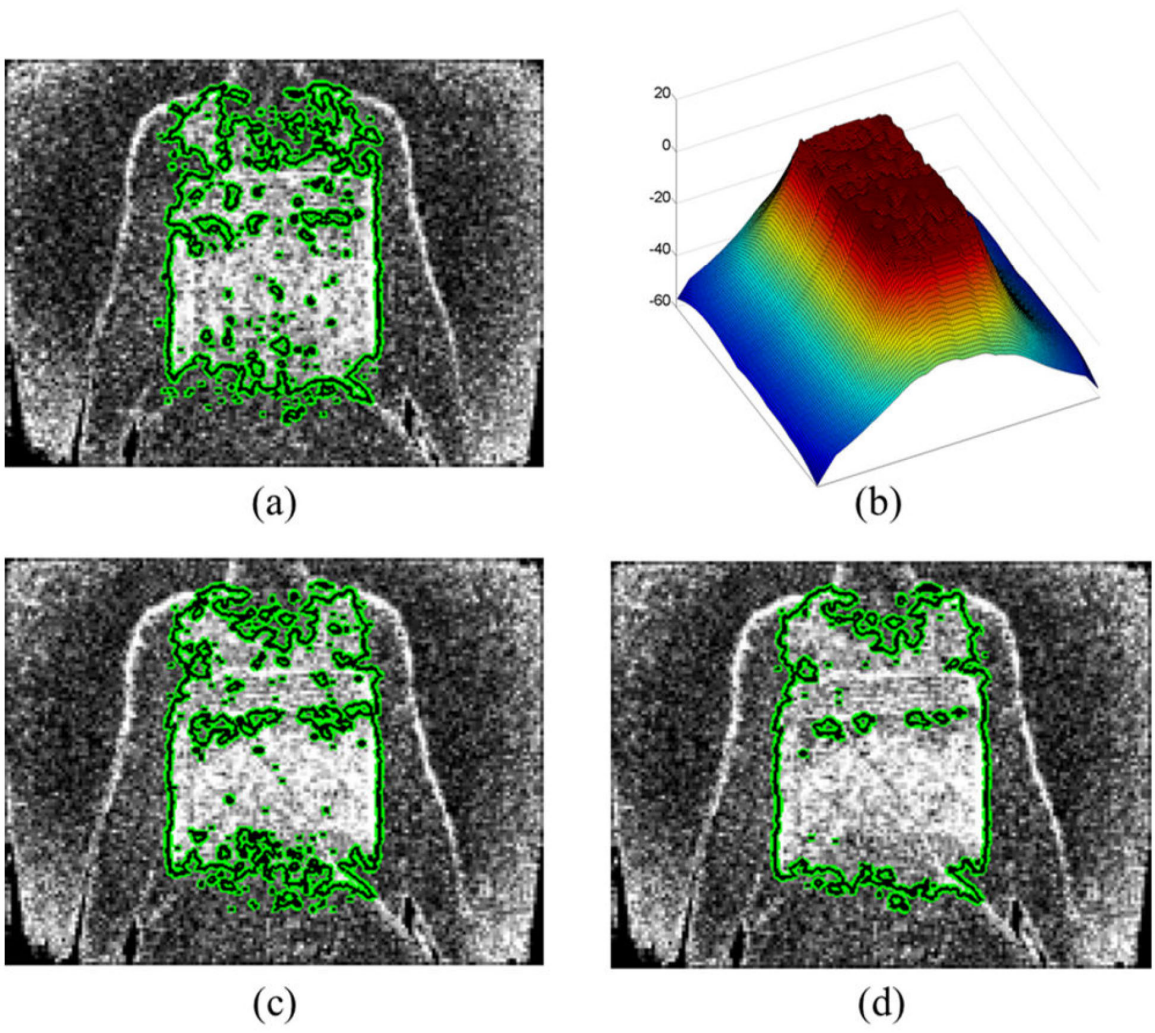
Segmentation result according to the use of the adaptive information function. (a) Segmentation result of the previous frame ${{\boldsymbol{R}}_{{\boldsymbol{t}} - 1}}$. (b) Visualization of adaptive information function. (c) Segmentation result without using adaptive information function. (d) Segmentation result using adaptive information function

An adaptive region information function was used to distinguish drastic changes in depth values due to noise or non-respiratory movement from those in the respiration-related region. This function was also used to include small changes in depth values due to respiration-related movement. This function takes the consecutive characteristics of the respiration into account, weights the respiration-related region from the previous frame to exclude the region changed by non-respiratory movement, and incorporates regions with little change into the respiration-related region. Fig. 6(a) represents the respiration-related region in the previous frame. The adaptive information function is affected by this region and it is similar to the region information function. However, unlike the region information function, the result of the previous segmentation is added as a weight to the chest wall region, as shown in Fig. 6(b). These weights ultimately affect the segmentation result. Fig. 6(c) and Fig. 6(d) show the segmentation result without or with the use of the adaptive information function. When not using this function, it can be seen that the small depth value changes in the abdominal region are excluded in the respiration-related region. However, when using the adaptive information function, it can be seen that the segmentation result was affected by the previous segmentation result, and that small depth value changes are included in the respiration-related region.

### D. Pulmonary Measurement Test

**Fig. 7.**
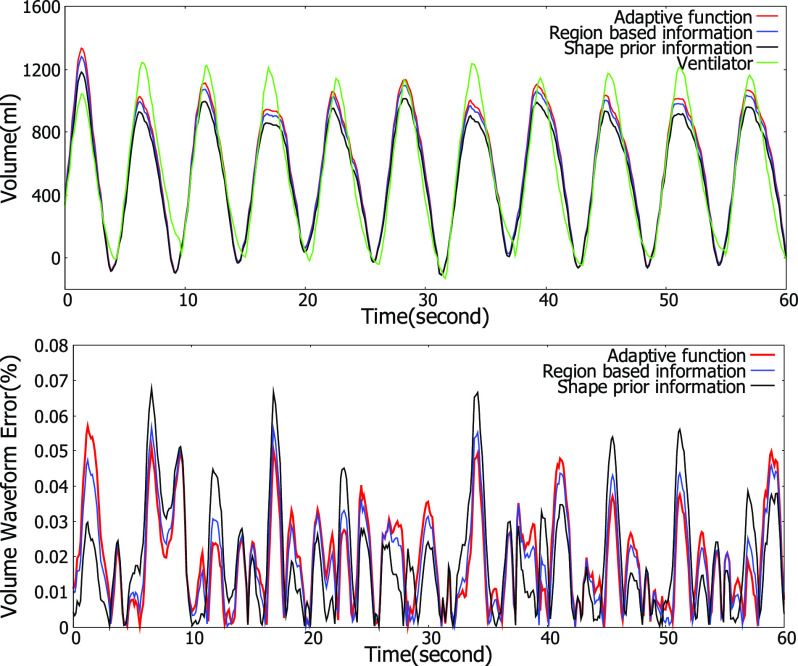
Respiratory volume waveform and waveform error obtained by different level set methods.

Methods presented in this paper were applied to measure the pulmonary function of 10 subjects. Subjects were instructed to exhale the air through a ventilator to perform actual breathing. In this test, the proposed method (level-set method with adaptive function) and other methods presented in this paper (level-set method with spatial information and level-set method with temporal information) were compared with the respiratory waveform and tidal volume calculated by a ventilator. The amount of respiratory input from the ventilator was set to 10 times, 15 times, and 20 times the predicted body weight (PBW) of the subject. Fig. 7 shows the respiratory volume waveform and waveform error obtained using the different methods. In the volume waveform, it can be seen that the volume waveform using level-set with the adaptive function method shows good agreement with the ventilator waveform. In addition, the volume waveform error is least at the peak and valley points, which is important when calculating the tidal volume. Table II shows the result of calculating the tidal volume error, volume waveform error, and intra-class correlation. Three subjects were excluded due to an abnormal ventilation leak and to body movement that was synchronized with breathing. In Table II, the mean tidal volume error was the smallest with the proposed method (8.41%), which is the level-set method with adaptive function. The mean respiratory volume waveform error was the smallest with the proposed method (25.09%). Moreover, the mean intra-class correlation showed the highest value with the level-set method with adaptive function ${\text{(ICC}} = {\text{0.893}})$. Mean values of the evaluation factors for the level-set method with spatial information were 14.34%, 35.92%, and 0.837, which include the highest error and the lowest correlation. Likewise, the mean values of the evaluation factors for the level-set method with temporal information were 10.93%, 29.59%, and 0.879. For most cases, the proposed method showed better performance in terms of tidal volume error, respiratory volume waveform error, and intra-class correlation than other methods did. As a result, it was confirmed that the proposed method calculated the tidal volume with smaller error, and that the intra-class correlation was also higher. Also, all methods showed differences in tidal volume error, respiratory volume waveform error, and intra-class correlation. The Kruskal-Wallis test was used to see if there was a statistically significant difference in the mean tidal volume error between various methods. Each sample used in the Kruskal-Wallis test consisted of the tidal volume error calculated for each breath in the volume waveform. Table III shows the result of the Kruskal-Wallis test. It was confirmed that there was a statistically significant difference between the methods for the mean tidal volume error ${\text{(p}} < {\text{0.0001}})$. Dunn's multiple comparison test was used to examine whether differences between groups were statistically significant. As a result, it was found that the differences between all the methods were statistically significant.

**TABLE II:** Comparing Evaluation Factors Between different Methods

Subject Information		Level-set method with spatial information			Level-set method with temporal information			Level-set method with adaptive function (proposed method)		
Subject No.	Input Volume (mm)	Tidal Volume Error (%)	Respiratory Volume Waveform Error (%)	Intra-class Correlation	Tidal Volume Error (%)	Respiratory Volume Waveform Error (%)	Intra-class Correlation	Tidal Volume Error (%)	Respiratory Volume Waveform Error (%)	Intra-class Correlation
2	780	10.49	18.39	0.946	9.12	14.74	0.968	10.13	14.05	0.965
	1200	15.57	24.73	0.900	9.38	19.02	0.942	5.49	12.44	0.965
	1500	17.31	25.45	0.888	14.03	21.86	0.916	8.9	14.88	0.958
3	730	16.38	38.19	0.808	7.36	22.40	0.925	6.68	26.51	0.881
	1100	28.01	35.91	0.768	15.93	22.94	0.900	6.49	17.51	0.938
	1500	10.22	22.37	0.907	9.46	14.68	0.955	9.63	14.69	0.955
5	780	14.11	38.42	0.844	7.54	21.21	0.934	8.29	22.86	0.915
	1200	18.11	39.03	0.817	13.77	25.72	0.909	11.51	18.67	0.948
	1600	14.26	48.02	0.763	13.70	38.14	0.794	4.45	28.83	0.872
6	680	11.68	39.12	0.820	11.34	42.40	0.805	11.89	40.18	0.795
	1000	15.43	38.42	0.839	13.83	36.35	0.853	11.56	35.05	0.863
	1400	12.54	68.66	0.625	11.85	66.47	0.677	11.34	62.1	0.633
7	650	14.63	67.24	0.698	13.75	62.60	0.720	9.71	38.78	0.844
	1000	15.85	43.93	0.829	11.37	36.86	0.859	9.91	34.94	0.868
	1300	10.85	35.68	0.842	8.30	27.39	0.883	7.89	29.01	0.913
8	720	17.83	37.18	0.824	7.63	23.30	0.920	5.95	18.43	0.958
	1100	10.07	30.50	0.880	8.64	22.27	0.924	6.73	16.12	0.951
	1400	10.34	19.70	0.928	9.76	16.27	0.953	6.7	11.96	0.969
9	730	18.67	30.51	0.871	15.29	29.11	0.886	6.33	19.48	0.946
	1100	9.98	19.54	0.934	8.79	22.43	0.922	8.57	20.59	0.91
	1500	8.82	33.25	0.834	8.67	35.19	0.822	7.5	35.13	0.81
Without Outlier	Mean	**14.34**	**35.92**	**0.837**	**10.93**	**29.59**	**0.879**	**8.41**	**25.09**	**0.893**
	STD	**4.29**	**13.14**	**0.076**	**2.70**	**13.69**	**0.076**	**2.16**	**12.12**	**0.081**

^*^STD: Standard deviation

**TABLE III:** Comparison of Mean Difference Between Groups Using Kruskal-Wallis Test

Groups	Mean rank		df	p-value		Kruskal-Wallis statistic
SP	1070.34		3	<0.0001		155.4
RB	856.15					
AR	703.01					
AR vs RB		AR vs SP			RB vs SP	
Yes		Yes			Yes	

^*^AR: Adaptive region method, RB: Region based method, SP: Shape prior method

### E. Dynamic Respiration-Related Region

**Fig. 8.**
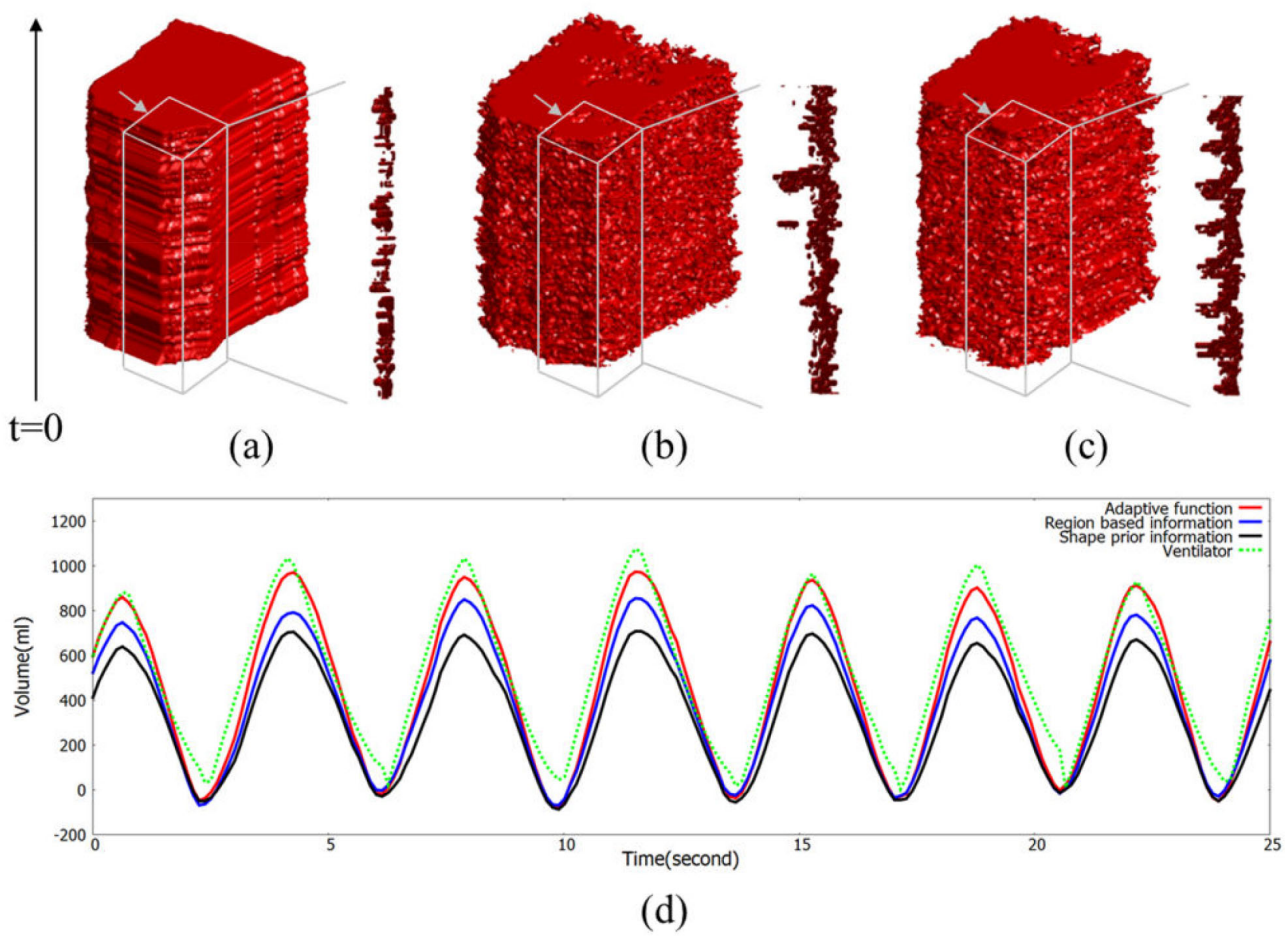
Visualization of dynamic changes in the respiration-related region over time among different methods. (a) Laminated respiration-related region using shape prior information. (b) Laminated respiration-related region using region based information. (c) Laminated respiration-related region using adaptive function. (d) Respiratory volume waveform based on the segmentation results of each method.

During respiration, the morphological changes of the chest wall occur as the air moves into and out of the lungs, increasing and decreasing the volume of the chest wall by the muscles involved in respiration. Fig. 8 shows dynamic changes in the respiration-related region and respiratory volume waveform according to each method. Fig. 8(a) shows the laminated respiration-related region and its cross section over time using shape prior information. In Fig. 8(a), it is hard to find a pattern that matches the cross section of the laminated

respiration-related region with the volume waveform in Fig. 8(d). Fig. 8(b) shows the laminated respiration-related region and its cross section over time using region-based information. In Fig. 8(b), the cross-sectional pattern of the laminated respiration-related region shows a pattern different from the pattern that appears in the cross section in Fig. 8(a). However, the pattern of the cross section of the laminated respiration-related region does not match the pattern of the respiratory volume waveform. Fig. 8(c) shows the laminated respiration-related region and its cross section over time using adaptive function. Unlike other methods, it can be seen that the respiratory volume waveform shows same pattern that appears in the cross section of the laminated respiration-related region. The variation of the respiration-related region according to the respiration pattern demonstrates that the proposed method has well separated morphological changes influenced by respiration. It can also be seen that the respiratory volume waveform calculated by the proposed method is closest to the respiratory volume waveforms of the ventilator.

## IV. Discussion

During respiration, there are morphological changes in the chest wall that include changes in the size of the chest wall region in the spatial domain and in the depth value in the time domain. Given this situation, the level-set method, combined with spatial and temporal information, was used to distinguish the change in the spatio-temporal region affected by respiration activity. As a result, a dynamic change in the size of respiration-related region was confirmed. Moreover, dynamic change in the respiration-related region was synchronized with the respiratory volume waveform pattern and demonstrated that the proposed method well-separated morphological changes influenced by respiration.

When modeling the adaptive function, a threshold of ‘1’ was set for all subjects to prevent repetitive addition of large weights to a specific region. This threshold was set empirically to a value that best segmented the respiration-related region when the level-set method was applied. The significant statistical difference between the two methods (adaptive region and region based) is due to the adaptive function. The level-set method with temporal information isolates the respiration-related region using the energy function which simply isolates the region exhibiting a certain depth difference in the chest wall region. However, the level-set method with adaptive function isolates the respiration-related region using the adaptive region information function. This function takes the consecutive effects from respiration into account, weights the respiration-related region from the previous frame to exclude regional changes caused by non-respiratory movement, and incorporates the parts of the region with little change into the respiration-related region. To calculate accurate tidal volume, all regions influenced by breathing should be included in the respiration-related region. Unlike the level-set method with temporal information, the adaptive region information function distinguishes regions where changes are caused by respiration, allowing calculation of tidal volume to be more accurate.

In the pulmonary measurement test, the experimental outliers were excluded and analyzed. Experimental outliers included cases where the respiratory volume waveform of a ventilator was not calculated accurately due to an abnormal ventilation leak, and the movements of the body synchronized with breathing were so severe that they interfered with the volume calculation.

Various ventilator input volumes were set in the pulmonary measurement test. These changes were to see if the accuracy of the measured tidal volume changed according to whether the input value was low or high. It was confirmed that there were significant linear relationships between BMI, vital capacity, and total lung capacity [17]. Because the vital capacity and total lung capacity varies from person to person, ventilator inputs were set at 10 times, 15 times, and 20 times the PBW. As a result, in Table II, there was no change in the accuracy of the measured tidal volume according to the various input tidal volumes. In addition, large input volume (20 times PBW) was tested to see if this technique could be used in a deep breathing trial. An incentive spirometer is used for deep breathing trials, but devices with mouth-pieces are difficult to use with tracheostomy patients. At the beginning of the design process for this study, it was appreciated that this technique might be used as an alternative to an incentive spirometer for tracheostomy patients.

The chest circumference of the subjects was in the range 80 to 96 cm and abdominal circumference was in the range 68 to 86 cm. No influence of subject chest or abdominal circumference on the tidal volume error could be found in the experimental results (Table II). Excluding the failed cases, the subject with the largest chest and abdominal circumference (Subject No. 8), as well as the subject with the smallest (Subject No. 7) had no particularly small or large tidal volume error compared to the other subjects. This was also true of the subject with the smallest abdominal circumference (Subject No. 3). Even if the chest and abdomen circumference had influence on the measurements, the affect would be so weak that it is unlikely to have a measurable impact on the calculated result of the tidal volume or respiratory volume waveform.

Depth images were recorded at 16 frames per second. In our experiments, subjects breathed once every 3–4 seconds. This is about 60 frames per respiration. Therefore, to process the algorithm, 60 frames of depth image per respiration are needed. The computational time required to produce the result of one respiration cycle (about 60 depth images) was about 603 seconds using an Intel Core i7-4770 CPU (3.40 GHz). This required computational time was mostly used to perform the level set method. The level-set method was performed in spatial domain and temporal domain. The level-set method using spatial information was performed in spatial domain (387 seconds) first. Using this result, the level-set method using adaptive information function was then performed in temporal domain (214 seconds). It took about 2 seconds to calculate the volume waveform. When using the conventional level-set method (Chan-Vese method), it took about 198 seconds to produce the result. Additional calculation time was required when performing the level-set method using spatial information because this method needs to calculate an energy term for the shape-prior knowledge.

## V. Conclusion

In this paper, a level-set segmentation method combined with spatial and temporal information was proposed to measure respiratory volume using a depth camera. The proposed method was evaluated by comparing tidal volume error, respiratory volume waveform error, and intra-class correlation with ventilator data. The proposed method was compared with two the other methods and the proposed method achieved higher accuracy in calculation of the evaluation factors than the other level-set methods did (proposed method: 8.41%, 25.09%, and 0.893; with spatial information only: 14.34%, 35.92%, and 0.837; with temporal information only: 10.93%, 29.59%, and 0.879). There was also a statistically significant difference ${\text{(p}} < {\text{0.0001}})$ between these methods. Therefore, it is confirmed that the proposed method calculates the respiratory volume more accurately than other methods do.
